# Herbal medicine use disclosure, database-flagged potential herb-drug interactions, and inter – interaction database concordance among patients with non-communicable diseases in Vietnam: A multicenter cross-sectional study

**DOI:** 10.1371/journal.pone.0355046

**Published:** 2026-07-31

**Authors:** Anh Dang Thuc Phan, Thi Ha Vo, Tram Nguyen Nguyet Luu, Hang Thu Thi Ngo, Michael Heinrich, Chuenjid Kongkaew

**Affiliations:** 1 Research Centre for Safety and Quality in Health, Faculty of Pharmaceutical Sciences, Naresuan University, Phitsanulok, Thailand; 2 Faculty of Pharmacy, University of Medicine and Pharmacy, Hue University, Hue, Vietnam; 3 Department of Pharmacy, Nguyen Tri Phuong Hospital, Ho Chi Minh, Vietnam; 4 Faculty of Pharmacy, Pham Ngoc Thach University of Medicine, Ho Chi Minh, Vietnam; 5 Research Group Pharmacognosy and Phytotherapy, UCL School of Pharmacy, University College London, London, United Kingdom; 6 Department of Pharmaceutical Sciences and Chinese Medicine Resources, Chinese Medicine Research Center, College of Chinese Medicine, Taichung, Taiwan; Kerman University of Medical Sciences, IRAN, ISLAMIC REPUBLIC OF

## Abstract

**Background:**

The widespread use of herbal medicines in clinical practice, particularly in Southeast Asia, presents significant challenges for medication safety management, notably regarding interactions between herbs and conventional drugs. Current drug interaction screening tools demonstrate substantial gaps in documenting herb-drug interactions, especially for regionally specific medicinal plants, creating potential risks for patients receiving concurrent therapies.

**Aim:**

(1) to identify the prevalence of potential herb–drug and herb–herb interactions and the disclosure rate of HM use in patients with NCDs; and (2) to assess the consistency of interaction information across commonly used drug interaction databases.

**Methods:**

An observational study enrolled 658 patients with non-communicable diseases from two Vietnamese tertiary care facilities. Structured interviews captured herbal medicine utilization patterns, disclosure practices, and specific botanical preparations used. Potential herb-drug and herb-herb interactions were systematically evaluated using four databases: Micromedex®, UpToDate Lexicomp Drug Interactions, Medscape Drug Interaction Checker, and Stockley’s Herbal Medicines Interactions. Inter-database agreement was assessed using Fleiss’ kappa statistics.

**Results:**

48.6% of participants reported active herbal medicines use, with 96% failing to disclose this to their healthcare providers. Potential interactions were identified in 31.3% of herbal medicine users, representing 15.2% of the total cohort. Database concordance was remarkably poor, with only 0.7% of interactions consistently documented across all four resources (Fleiss’ κ = –0.0653; p < 0.001). Multiple indigenous medicinal plants commonly used by patients were absent from all evaluated databases.

**Conclusion:**

The substantial prevalence of undisclosed herbal medicine use, alongside inadequate representation in drug interaction databases, represents a critical gap in medication safety infrastructure. These findings demonstrated the necessity for integrating herbal medicine assessment into routine care, enhanced provider training, and expansion of clinical decision support systems to include region-specific botanical data for populations using concurrent traditional and conventional therapies.

## Introduction

The integration of herbal medicine (HM) into clinical practice poses growing medication safety challenges, particularly among patients with non-communicable diseases (NCDs), who commonly use HMs alongside long-term pharmacotherapy [1]. In healthcare systems such as Vietnam’s, where HM use is widespread and institutionally recognized [2], concurrent use with conventional medicines has direct implications for clinical decision-making and patient safety. A central concern arising from this practice is the risk of clinically significant herb–drug interactions (HDIs), which may compromise therapeutic efficacy or precipitate adverse events, especially for medications with narrow therapeutic indices [3,4].

Despite increasing awareness of HDI-related risks, reliable identification and management remain difficult in routine care. Existing evidence is structurally limited by heterogeneity in herbal product composition, insufficient pharmacokinetic and mechanistic data, and incomplete documentation of real-world use [5,6]. Among these challenges, patient non-disclosure of HM use represents a critical barrier, undermining medication reconciliation and restricting clinicians’ ability to anticipate, detect, and manage interaction-related harms [4,7].

Although systematic HDI data collection is essential to support safe prescribing, the current evidence base remains fragmented and methodologically inconsistent, relying largely on isolated case reports and poorly documented observations [8]. In response, multiple herb–drug interaction databases have been developed as clinical decision-support tools [9–15]. However, substantial variability across these platforms in the identification, classification, and grading of interactions raises concerns regarding their reliability and their role in supporting consistent clinical risk assessment [15].

Accordingly, this study aimed to: (1) identify the prevalence of potential herb–drug and herb–herb interactions and the disclosure rate of HM use in patients with NCDs; and (2) assess the consistency of interaction across commonly used drug interaction databases.

## Materials and methods

### Setting and participants

A cross-sectional study was conducted from 31 August 2024 to 31 January 2025 among outpatients with NCDs at two Grade I general hospitals in central and southern Vietnam. Before initiating data collection, the principal investigator organized a one-hour training session for clinical pharmacists from both hospitals to standardize interview procedures and ensure uniformity in data collection practices. Between 31 August 2024 and 31 October 2024, the research team conducted face-to-face patient interviews at the hospital located in southern Vietnam. Subsequently, data collection proceeded at the hospital in central Vietnam from 03 November 2024 to 31 January 2025.

Eligible participants were adults (≥18 years) with confirmed NCD diagnoses (ICD-10 codes) who provided written informed consent. Individuals unable to respond to the interviewer’s questions were excluded.

### Ethical considerations

The study was conducted in accordance with the Declaration of Helsinki. Ethical approval was obtained from Naresuan University Institutional Review Board (P1-0106/2567), and two research sites (H2024/371; 1743/NTP-HĐĐĐ). No individual community leaders provided approval, as the study was hospital-based. Prior to any study-related activities, each patient was provided with written informed consent. Participant confidentiality was maintained through the anonymization of all data, whereby names and clinic record numbers were omitted from all study forms.

### Inclusivity in global research

Additional information regarding the ethical, cultural, and scientific considerations specific to inclusivity in global research is included in the S1 Checklist.

### Sample size

This study calculated the sample size for a prevalence study, the formula commonly applied is based on estimating a proportion. The standard formula is as follows [16]:


$$\begin{array}{c} n=\;\frac{Z^{\text{2}}x\;p\;x\;(\text{1}-p)}{d^{\text{2}}} \end{array}$$

where Z represents the z-score corresponding to the desired confidence level (z = 1.96 with 95% confidence level), p is the estimated prevalence (p = 0.5) following a previous study [2], d is the desired margin of error (d = 0.05). Accounting for a 30% non-response rate, the minimum sample size was 500 participants across two hospitals. The sample size in this study was determined by the primary prevalence objective, not by the predefined secondary analyses.

### Data collection

Outpatients with at least one NCD were recruited through random sampling at both study sites. Daily eligible patient lists from outpatient departments were assigned identification numbers, with participants selected via computer-generated randomization. Pharmacists identified selected patients in the dispensing area and invited participation. Following study explanation and written informed consent, interviews were conducted in private rooms.

### Questionnaire

HMs in this study were defined as crude herbs; or plant-based supplements, often poorly defined chemically preparations from herbalists; or patient-made remedies from markets or home gardens [17]. To improve comprehension, a reference list of commonly used herbs and herbal products was provided [2]. Participants were also instructed to report all over-the-counter products, supplements, and frequently consumed foods at the time of completing the questionnaire. Local plant names reported by participants were first documented verbatim and then cross-checked using Vietnamese pharmacopeia references and authoritative local herbal medicine texts. Where possible, names were further verified by pharmacists with training in traditional medicine, who assessed consistency between vernacular names and plant parts used. Herbs that could not be confidently matched to an accepted botanical name after this process were classified as unidentified and excluded from database-based interaction analyses.

The questionnaire, adapted from the International *Questionnaire to Measure Use of Complementary and Alternative Medicine (I-CAM-Q)* [18] and translated into Vietnamese by the principal investigator (ADTP) – an origin Vietnamese with a qualified English certificate. The draft Vietnamese version was reviewed to ensure conceptual equivalence with the original instrument and contextual appropriateness for Vietnamese clinical settings. The final questionnaire comprised three sections: (1) sociodemographic characteristics; (2) clinical characteristics, including type and duration of non-communicable diseases (NCDs) and current medications; and (3) HM use patterns, including types of products used, frequency of use, sources of procurement, reasons for use, and disclosure to healthcare providers (S2 File).

Content validity was assessed through expert evaluation using the Item–Objective Congruence (IOC) method [19]. The expert panel consisted of five specialists: three clinical pharmacists with experience in medication safety and two herbal medicine specialists with expertise in traditional medicine practice. Each item was independently rated for relevance and clarity. All questionnaire items achieved IOC values above the predefined acceptability threshold of 0.75, with scores ranging from 0.80 to 1.00, indicating good content validity.

To assess clarity and feasibility, the questionnaire was pilot-tested with 10 patients representative of the target study population. Feedback from the pilot testing indicated that the items were generally clear and understandable, and minor wording refinements were made to improve comprehension. Data from the pilot phase were not included in the final analysis.

### Potential interactions assessment

Potential interactions, including potential herb-drug (pHDIs) and herb-herb interactions (pHHIs), were identified using a modified drug-drug interaction definition: pharmacological or clinical responses to combinations differing from expected individual effects, potentially causing antagonistic, synergistic, or idiosyncratic outcomes [20]. Four tertiary databases: UpToDate Lexicomp Drug Interactions [13], Micromedex® [10], Medscape Drug Interaction Checker [11], and Stockley’s Herbal Medicines Interactions [9] were searched to minimize missed interactions. All reported HMs and prescription drugs were screened across databases, documenting interaction severity, evidence quality, and management recommendations.

#### Analytic units and pairing rules.

Three units of analysis were used throughout the study and were distinguished consistently:

(i) the patient (denominators: n = 658 for the full cohort; n = 320 for HM users);
(ii) the unique pair: a single herb paired with a single co-administered conventional drug (pHDI) or with another reported herb (pHHI), counted once per patient regardless of how many databases flagged it;
(iii) the interaction occurrence: a unique pair as identified by a single database, such that one pair flagged by three databases contributed three occurrences to the database-concordance analysis.

For each HM user, every reported herb was paired with every concurrently used prescription drug and with every other reported herb. Multi-ingredient herbal products were decomposed into individual constituents, and each constituent was paired separately. Duplicate prescription drugs (i.e., the same agent appearing on more than one prescription line for the same patient) were counted once per patient. A single patient could therefore contribute multiple unique pairs and multiple interaction occurrences; conversely, the same herb–drug pair contributed by different patients was counted separately at the patient level but pooled when reporting unique pairs at the database-evaluation level.

#### Severity grading.

Severity for each occurrence was extracted using each database’s native grading system and harmonized to a common five-level scale (none, minor, moderate, major, severe) following Lexicomp’s framework (Table 1) [21,22]. HMs reported by participants but absent from all four databases were classified as “not evaluable” and were excluded from the interaction-prevalence and concordance analyses.

**Table 1:** Classification of potential interactions by severity amongst drug interaction databases.

	Lexicomp	Micromedex	Medscape	Stockley’s herbal medicine interaction	
**Severe**	(X) Avoid combination	Contraindicated	Contraindicated	Contraindicated	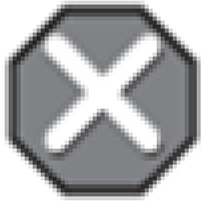
**Major**	(D) Consider therapy modification	Major	Serious – Use alternative	Severe	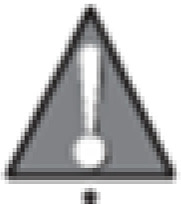
**Moderate**	(C) Monitor therapy	Moderate	Monitor Closely	Moderate	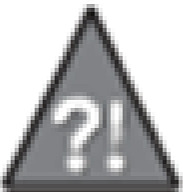
**Minor**	(B) No action needed	Minor	Minor	Mild	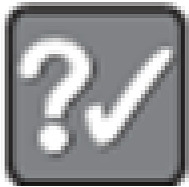
**None**	(A) No known interaction	Unknown	No interactions found	Nothing expected	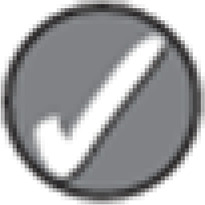

### Outcomes

The primary outcome was the prevalence of potential interactions involving medicinal plants and conventional drugs or among multiple medicinal plants. This was calculated at the patient level as the proportion of HM users with at least one identified interaction relative to the total number of HM users. Secondary outcomes included rates of non-disclosure of HM use, and the level of agreement among the four databases in classifying interaction severity, with greater agreement reflecting higher consistency across databases.

### Data analysis

Patient-level outcomes, including patient characteristics, non-disclosure rates, and the prevalence of potential interactions, were summarized using descriptive statistics, with the full cohort (n = 658) or HM users (n = 320) serving as the denominators, as appropriate. Pair-level descriptive statistics were calculated using the total number of interaction occurrences (n = 307) as denominator. The specific denominator applied was explicitly stated in each table and figure legend.

Inter-database agreement was evaluated using Fleiss’ kappa, with the four databases treated as “raters” and each unique interaction pair (n = 120) considered the “subject” rated according to the five-level harmonized severity scale. Kappa statistics were calculated both overall and separately for the minor, moderate, and major severity categories. A negative κ value indicates agreement lower than that expected by chance, suggesting discordance among databases regarding whether and how a given interaction pair should be classified. Both negative and positive κ values were interpreted in accordance with the criteria proposed by Landis and Koch (<0.0: poor; 0.0–0.2: slight; 0.21–0.40: fair; 0.41–0.60: moderate; 0.61–0.80: substantial; 0.81–1.00: almost perfect) [23]. Database overlap was additionally quantified as the proportion of each unique pair appearing in one, two, three or four databases, using total unique pairs (n = 120) as the denominator. Statistical significance was set at p < 0.05. Kappa values were computed in RStudio using the *irr* package (v 0.84.1) [24]. All other analyses were performed in SPSS v 25.0.

## Results

### Participant characteristics

**Fig 1:**
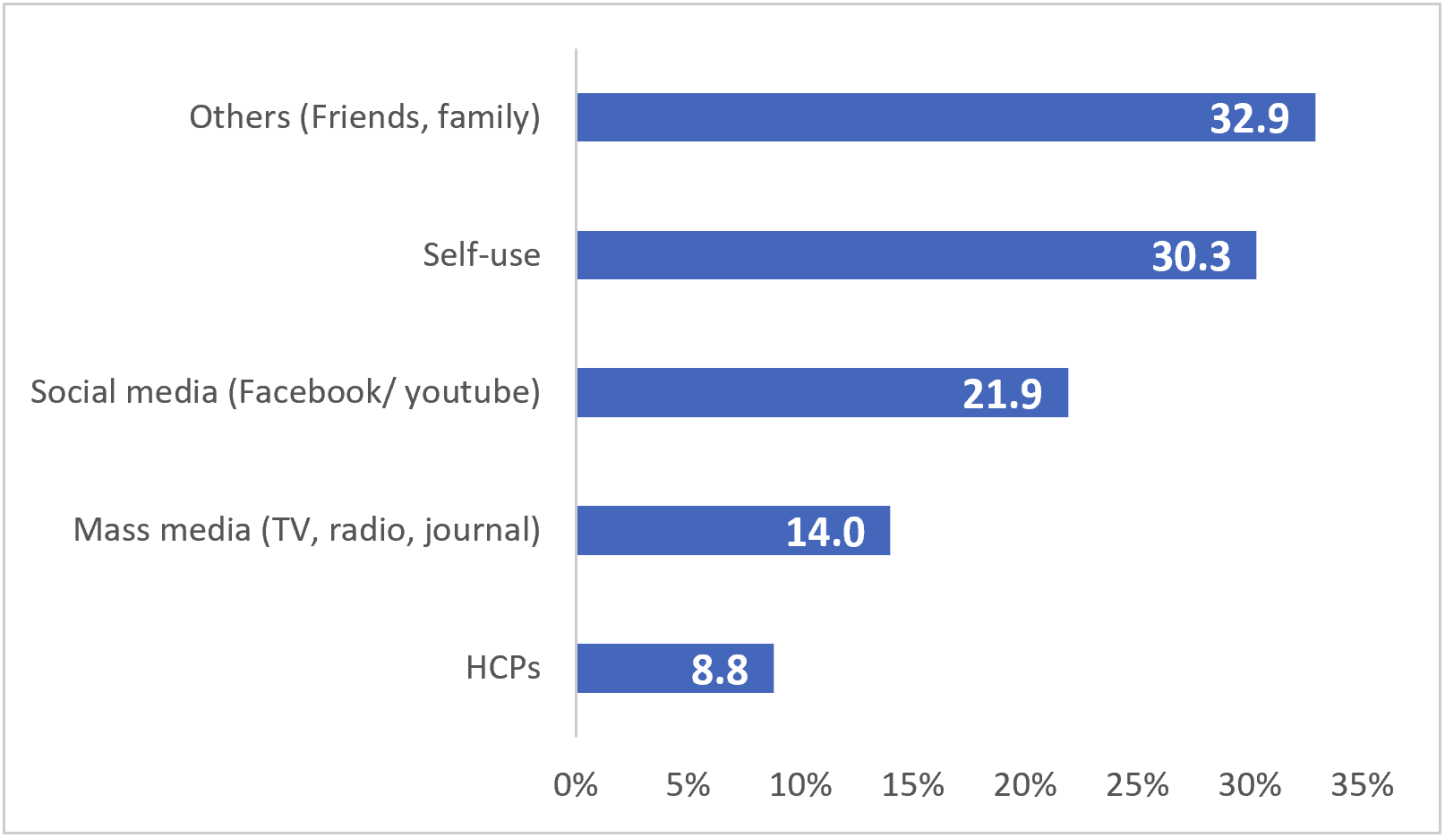
Sources of HMs information.

**Fig 2:**
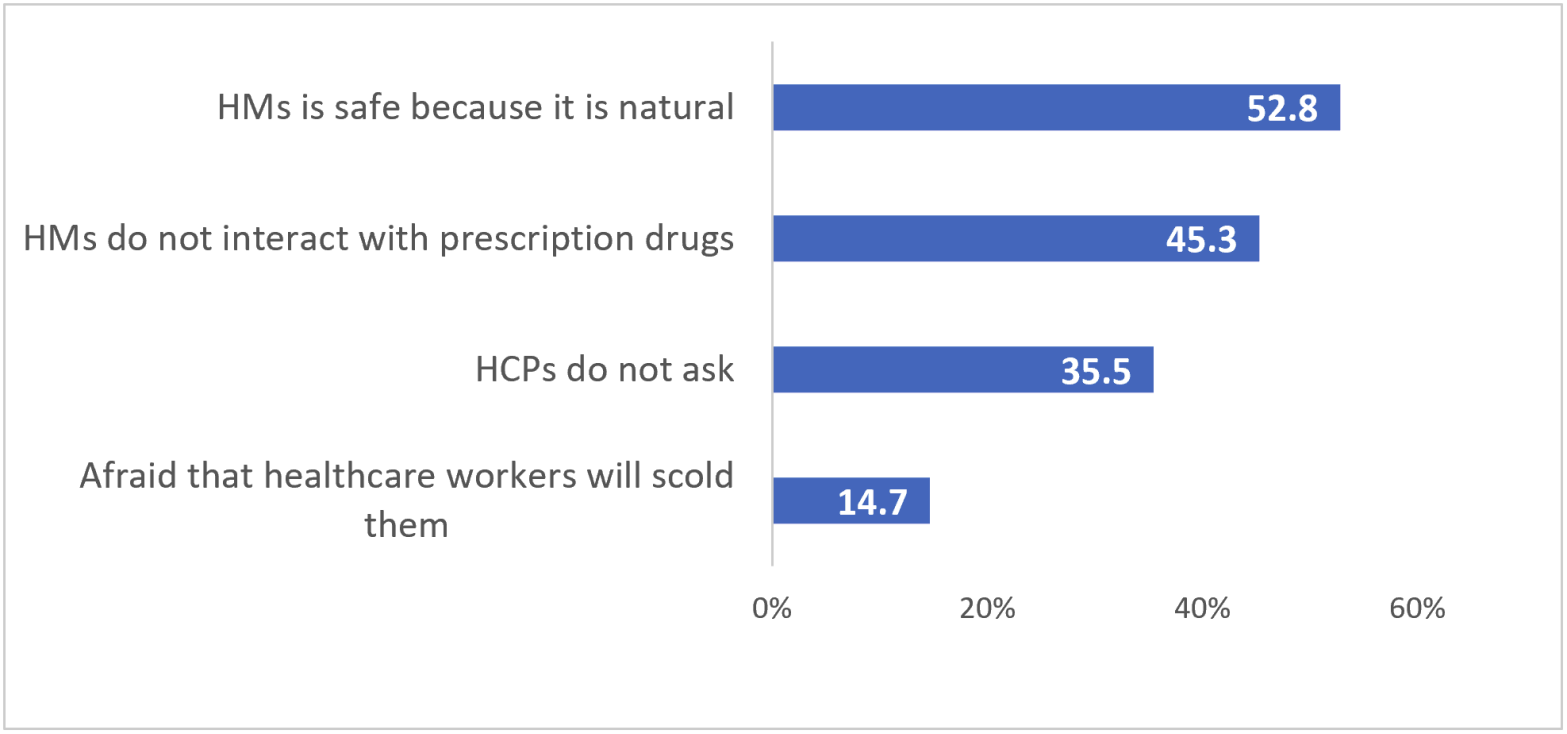
Reasons for non-disclosure to HCPs.

Of 800 individuals invited, 658 completed interviews (82.3% response rate). Most participants were female (67.0%), and more than half were aged ≥ 60 years (65.3%). Over half were unemployed (54.6%); most lived in urban areas (77.7%). Self-rated health was fair (46.8%), good (34.7%), or poor (18.5%). Educational attainment was low: 65.5% had not completed high school, 3.2% never attended school. Multimorbidity was common: 33.1% had 2–3 chronic conditions, 39.5% had more than three, primarily cardiovascular disease, hypertension, diabetes, and hyperlipidemia. Polypharmacy (>5 medications) occurred in 48.9%. Nearly half of respondents (n = 320, 48.6%) reported current use of HMs at the time of the interview (Table 2). Information sources included friends/family (32.9%), self-initiated use (30.3%), social media (21.9%), and healthcare professionals (8.8%) (Fig 1). Among HM users, the vast majority of respondents (n = 307, 96%) reported not disclosing their use of HMs to HCPs. Reasons for non-disclosure included beliefs that “HM is safe because it is natural” (52.8%), no perceived interactions with conventional medicines (45.3%), lack of provider inquiry (35.5%), and fear of disapproval (14.7%) (Fig 2).

**Table 2:** Characteristics of participants (N = 658).

Characteristics	Frequency	Percent
**Age group (year)**		
< 60	228	34.7
≥ 60	430	65.3
**Gender**		
Male	217	33.0
Female	441	67.0
**Living area**		
Rural	147	22.3
Urban	511	77.7
**Educational level**		
Not going to school	21	3.2
Less than high school	431	65.5
More than high school	206	31.3
**Employment**		
No	359	54.6
Yes	299	45.4
**Self-perceived health status**		
Poor	122	18.5
Fair	308	46.8
Good	228	34.7
**Smoking**		
No	536	81.5
Yes	122	18.5
**Doing exercise**		
No	280	42.6
Yes	378	57.4
**Drinking alcohol**		
No	571	86.8
Yes	87	13.2
**Number of Comorbidities**		
< 2	180	27.4
2-3	218	33.1
> 3	260	39.5
**Cardiovascular conditions**		
No	448	68.1
Yes	210	31.9
**Hypertension**		
No	285	43.3
Yes	373	56.7
**Diabetes Mellitus**		
No	374	56.8
Yes	284	43.2
**Hyperlipidemia**		
No	357	54.3
Yes	301	45.7
**Hepatic diseases**		
No	588	89.4
Yes	70	10.6
**Kidney diseases**		
No	589	89.5
Yes	69	10.5
**Arthritis**		
No	561	84.3
Yes	97	14.7
**Cancer**		
No	643	97.7
Yes	15	2.3
**COPD/ Asthma**		
No	628	95.4
Yes	30	4.6
**Thyroid disease**		
No	560	85.1
Yes	98	14.9
**Allergy history**		
No	621	94.4
Yes	37	5.6
**Number of prescription drugs**		
≤ 2	178	27.1
3 - 4	158	24.0
≥ 5	322	48.9
**HM use**		
Yes	320	48.6
No	338	51.4
**Disclosure to HCPs (n = 320)**		
Yes	13	4.0
No	307	96.0

### Potential herb-drug interactions amongst patients with NCDs

#### Patient-level burden.

Of the 320 HM users, 100 (31.3%) had at least one potential interaction identified in one or more databases, corresponding to 15.2% of the full cohort (100/658). Among these 100 patients, the number of unique interaction pairs per patient ranged from 1 to 14, with a median of 2 (IQR = 2), indicating that individual patients frequently contributed several pairs to the pair-level analysis.

#### Pair-level burden.

Among evaluable HM users, the four interaction databases collectively identified 307 potential interaction occurrences, comprising 120 unique interaction pairs (105 unique pHDI pairs and 15 unique pHHI pairs). Medscape identified the greatest number of interactions (120 occurrences across 51 unique pairs), followed by Lexicomp (94 occurrences across 33 pairs), Micromedex (64 occurrences across 24 pairs), and Stockley’s (29 occurrences across 12 pairs). Only Medscape (27 interaction occurrences involving 14 unique pairs) and Lexicomp (1 interaction occurrence involving 1 unique pair) identified pHHIs (S3 and S4 Tables). Overall, 279 of the 307 interaction occurrences were pHDIs, whereas 28 were pHHIs. Following harmonization to a common severity classification, 63.5% (195/307) of interaction occurrences were classified as moderate, 32.6% (100/307) as minor, and 3.9% (12/307) as major. No pHHI was classified as major (Table 3).

**Table 3:** Severity distribution of potential interactions across drug interaction databases.

Databases	Minor		Moderate		Major		Total
	pHDIs	pHHIs	pHDIs	pHHIs	pHDIs	pHHIs	
Lexicomp	39	0	53	1	1	0	**94**
Micromedex	1	0	52	0	11	0	**64**
Medscape	31	3	62	24	0	0	**120**
Stockley’s	26	0	3	0	0	0	**29**
**Total**	**97**	**3**	**170**	**25**	**12**	**0**	**307**

The medicinal plants most frequently involved across the 307 occurrences were green tea (24.4%, 75/307), bitter melon (9.8%, 30/307), dong quai (6.8%, 21/307), ginseng (6.8%, 21/307), and licorice (6.5%, 20/307) (Table 4). The prescription drugs most often implicated were atorvastatin (13.7%, 42/307), clopidogrel (12.4%, 38/307), metformin (9.1%, 28/307), insulin (5.2%, 16/307), and aspirin (4.6%, 14/307) (Table 5).

**Table 4:** Top medicinal plants related to potential interactions.

Common names	Scientific names	n	Percent*
Green Tea	*Camellia sinensis* (L.) Kuntze	75	24.4
Bitter Melon	*Momordica charantia* L.	30	9.8
Dong quai	*Angelica sinensis* (Oliv.) Diels	21	6.8
Ginseng	*Panax ginseng* C.A. Meyer	21	6.8
Licorice	*Glycyrrhiza glabra* L.	20	6.5
Cinnamon	*Cinnamomum verum* J. Presl	13	4.2
Turmeric	*Curcuma longa* L.	13	4.2
Ginkgo biloba	*Ginkgo biloba* L.	11	3.6
Mistletoe	*Viscum album* L.	8	2.6
Sage	*Salvia officinalis* L.	8	2.6
Reishi	*Ganoderma spp.*	2	0.7
Saw palmetto	*Serenoa repens* (W. Bartram)	2	0.7
Garlic	*Allium sativum* L.	1	0.3
Other medicinal plants		82	26.7
**Total**		**307**	**100.0**

*Percentages are expressed relative to 307 interaction occurrences. A single patient could contribute to multiple occurrences.

**Table 5:** Popular prescription drugs related to potential interactions.

ATC code	Drugs	n	Percent*
C10AA05	Atorvastatin	42	13.7
B01AC04	Clopidogrel	38	12.4
A10BA02	Metformin	28	9.1
A10AB01	Insulin	16	5.2
B01AC06	Aspirin	14	4.6
C07AB07	Bisoprolol	12	3.9
C08CA01	Amlodipine	10	3.3
A10BH01	Sitagliptin	6	2.0
N02BE01	Acetaminophen	5	1.6
C09CA01	Losartan	5	1.6
C07AB02	Metoprolol	4	1.3
C03AA03	Hydrochlorothiazide	3	1.0
A10BB09	Gliclazide	2	0.7
C09AA04	Perindopril	2	0.7
C09CA07	Telmisartan	2	0.7
Other drugs		118	38.4
**Total**		**307**	**100.0**

*Percentages were expressed relative to 307 interaction occurrences. A single patient could contribute to multiple occurrences.

### Data concordance and coverage gaps

Fleiss’ kappa, computed across all 120 unique pairs with the four databases as raters and the five-level harmonized severity scale as the rating categories, indicated poor overall agreement (κ = –0.065; p < 0.001). Category-specific κ values were also negative and statistically significant for the minor (–0.146; p = 0.006) and moderate (–0.172; p < 0.001) categories, and negative but non-significant for the major category (–0.266; p = 0.119) (Table 6).

**Table 6:** Kappa agreement indices among four drug interaction databases.

Category	Fleiss Kappa	P value
Minor	−0.146	0.006
Moderate	−0.172	<0.001
Major	−0.266	0.119
Overall	−0.0653	<0.001

**Fig 3:**
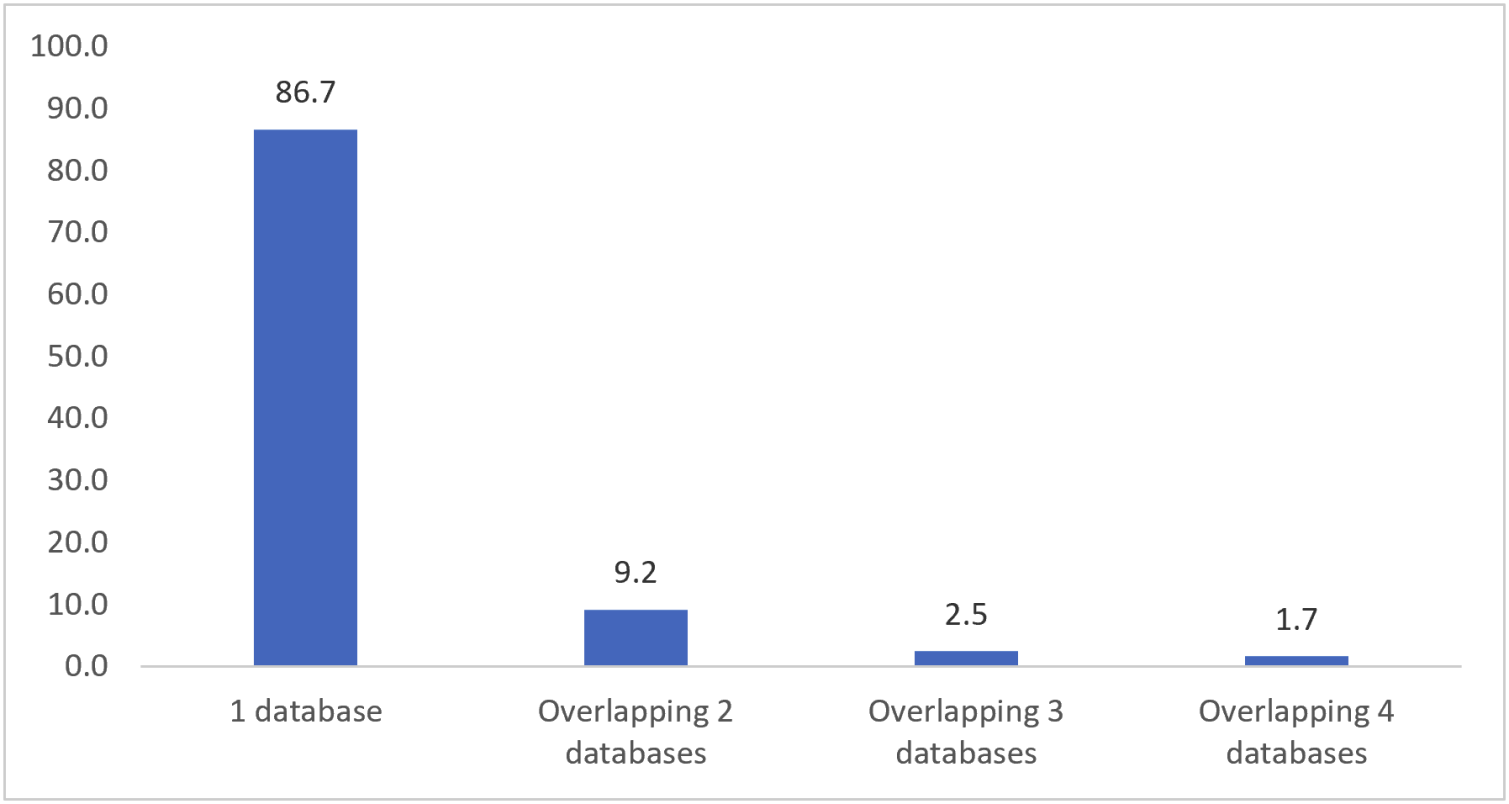
Overlap of potential interactions across four databases.

Consistent with the low degree of database concordance, overlap across databases was minimal. Of the 120 unique pairs, only 2 (1.7%) were identified by all four databases, 3 (2.5%) by three databases, and 11 (9.2%) by two databases, whereas the vast majority (104 pairs, 86.7%) were identified by only one database (Fig 3).

#### Coverage of locally used medicinal plants.

During database screening, 135 HM users (42.2% of 320 HM users) could not be evaluated for potential interactions because one or more of the medicinal plants they reported were absent from all four databases. The most frequently missing plants were *Syzygium nervosum* DC. [leaves] (12.6%, 17/135), *Pandanus amaryllifolius* Roxb. [leaves] (11.1%, 15/135), *Polyscias fruticosa* (L.) Harms [whole plant] (6.7%, 9/135), *Carica papaya* L. [leaves/flowers] (6.7%, 9/135), and *Ficus racemosa* L. [leaves] (5.9%, 8/135) (Table 7).

**Table 7:** Top local medicinal plants unrepresented in all databases.

Local name of medicinal plant	Scientific name of herb	Part of plant used	n	Percent*
Vối	*Syzygium nervosum* DC.	Leaf	17	12.6
Lá dứa	*Pandanus amaryllifolius* Roxb.	Leaf	15	11.1
Đinh lăng	*Polyscias fruticosa* (L.) Harms	Whole plant	9	6.7
Lá/ hoa đu đủ	*Carica papaya* L.	Leaf and flower	9	6.7
Sung	*Ficus racemosa* L.	Leaf	8	5.9
Đậu đen	*Phaseolus vulgaris* L.	Seed	6	4.4
Diệp hạ châu	*Phyllanthus urinaria* L.	Whole plant	5	3.7
Thục địa	*Rehmannia glutinosa* (Gaertn.) Libosch.	Root	3	2.2
Mã đề	*Plantago major* L.	Leaf	3	2.2
Xạ đen	*Ehretia asperula* Zoll. et Mor.	Leaf	3	2.2
Cà gai leo	*Solanum procumbens* Lour.	Whole plant	2	1.5
Mật gấu	*Vernonia amygdalina* Del.	Leaf	2	1.5
Đậu bắp	*Abelmoschus esculentus* (L.) Moench	Immature green pods	2	1.5
Sen	*Nelumbo nucifera* Gaertn.	Leaf	2	1.5
Other medicinal plants			49	36.3
**Total**			**135**	**100.0**

*Percentages were expressed relative to the 135 HM users for whom one or more reported plants were absent from all four databases.

## Discussion

This study demonstrated that the concurrent use of HMs among patients with NCDs was common, while disclosure to healthcare providers was exceptionally rare. This combination represented a critical medication safety vulnerability in healthcare systems where traditional medicine is culturally embedded and widely accessible. The finding that nearly half of participants used HMs, yet fewer than 5% disclosed such use, underscored a systemic rather than individual failure.

Patient explanations for non-disclosure in this study aligned closely with established communication research, which conceptualized disclosure as a context-dependent behavior shaped by beliefs, expectations, and interactional cues rather than deliberate concealment [25,26]. The widespread perception that “natural equals harmless” reflects a cognitive framing that positions HMs outside the domain of “medication,” thereby rendering disclosure unnecessary from the patient’s perspective. This mirrors prior findings in which patients omitted complementary medicine use because it was perceived as intrinsically safe or irrelevant to biomedical care [7,26]. Clinician behaviour reinforced this pattern: many participants reported that HM use was never raised during consultations, and patients appeared to interpret this silence as tacit confirmation that such use was not relevant to disclose [5,27]. Non-disclosure is therefore best understood not as patient non-adherence, but as an interactional outcome co-produced by patient beliefs and a clinical communication environment that fails to invite or normalise such discussions [26]. Notably, participants were more willing to disclose HM use to clinical pharmacists than to physicians or nurses, consistent with models of person-centred, collaborative communication that emphasise time, attentiveness, and shared problem-solving as facilitators of disclosure [27,28]. Given physicians’ time constraints and pharmacists’ established role in medication reconciliation, pharmacists are well placed to act as accessible gatekeepers for HM screening; embedding structured HM inquiry within pharmacist-led consultations, in line with international recommendations [7,29,30], may be a pragmatic strategy to improve disclosure rates in high-volume clinical settings.

Beyond communication, this study identified a substantial burden of interaction in patient-level prevalence (31.3% of HM users) and pair-level burden (307 interaction occurrences across 120 unique pairs). While prevalence estimates varied across countries, likely reflecting differences in herbal practices, populations, and database methodologies [31,32], the clinical implications remain significant. HMs contain multiple bioactive phytochemicals that share metabolic and pharmacodynamic pathways with conventional drugs, rendering interaction risks mechanistically plausible and clinically relevant [33]. In patients with NCDs and polypharmacy, even interactions classified as “moderate” may lead to therapeutic failure or adverse outcomes, particularly in older or vulnerable individuals [33,34]. The absence of standardized HDI guidelines further complicates risk stratification, supporting calls for routine interaction screening in multimorbid populations [35]. Importantly, the identification of pHHIs in 10% of participants highlighted that safety concerns extend beyond conventional drug co-use. Polyherbal combinations observed in this study raise phytovigilance concerns, as synergistic or antagonistic effects may occur in the absence of prescriber oversight [36].

A critical system-level finding was the marked inconsistency across drug-interaction databases, reflected in a negative overall Fleiss’ κ – that is, the four resources agreed on the existence and severity of a given pair less often than would be expected if they had been classifying pairs at random. This variability exposes a structural limitation: interaction databases are often treated as authoritative decision-support tools, yet their outputs depend heavily on non-standardized inclusion criteria, evidence thresholds, and severity grading systems [37]. Inconsistent reporting risks both over-alerting and under-detection, contributing to alert fatigue and potentially undermining clinician confidence in safety systems. The near absence of HHI data in most databases further illustrates a pharmacovigilance blind spot, particularly in regions where polyherbal use is common. While platforms such as Medscape identified more potential interactions, especially HHIs, the limited depth of documentation highlights the trade-off between accessibility and evidentiary rigor [38,39]. These findings underscored that databases cannot compensate for incomplete medication histories or poor product documentation. More fundamentally, the lack of detailed information on herbal product composition represented a persistent challenge for both clinical interpretation and signal detection. Adulteration, variable quality, and undeclared ingredients are well-recognized global concerns, especially where products are regulated as foods or supplements rather than medicines [40,41]. Without standardized reporting of preparation composition, attributing causality in suspected interactions remains problematic, reinforcing calls for enhanced phytovigilance and regulatory oversight [38,40].

Taken together, these findings indicated that HDI risk in this setting arose not only from pharmacological mechanisms but from gaps in disclosure, clinical communication, and the reliability of existing decision-support tools. Addressing these gaps required closing the communication loop between patients and providers, drawing on pharmacists’ position in medication review, and developing region-specific, harmonised phytovigilance data to support safer concurrent use of herbal and conventional medicines.

Strengths of this study included the large and clinically diverse cohort of patients with NCDs, enhancing the relevance of the findings to routine care. HM use was comprehensively ascertained using a standardized questionnaire (I-CAM-Q) and collected through face-to-face interviews conducted by trained clinical pharmacists, which likely improved data completeness and disclosure. The comparative assessment across multiple widely used drug interaction databases allowed for systematic evaluation of consistency and variability in identified herb–drug and herb–herb interactions. Together with expert content review and the population-based sampling approach, these strengths provide a robust and practice-relevant overview of potential interaction risks in real-world clinical settings. However, several limitations should be noted. HMs are heterogeneous, and limited information on product composition, quality, dose, and duration restricted causal attribution and assessment of exposure-dependent interaction risk. Identified herb–drug and herb–herb interactions were database-derived and therefore theoretical, reflecting potential rather than clinically confirmed harm. HM use was self-reported and subject to recall bias, which may have led to underestimation of use and associated risks. Finally, as the study was conducted in a Southeast Asian context where traditional medicine is culturally embedded, differences in use patterns, regulation, and healthcare communication may limit generalisability to other settings.

## Conclusion

Undisclosed HM use was common among patients with NCDs and was associated with a substantial burden of potential herb–drug and herb–herb interactions, representing an under-recognized medication safety risk in routine care. Inconsistencies and gaps across interaction databases, particularly for regionally used medicinal plants, further limited reliable risk assessment. These findings underscored the need for systematic integration of HM screening into medication reconciliation, strengthened phytovigilance and regulatory oversight, pharmacist-led counselling to improve disclosure, and decision-support systems incorporating transparent, region-specific herbal data. Coordinated action across clinical practice, policy, and digital health was essential to support the safe co-use of herbal and conventional medicines.

## Supporting information

S1 Checklist Questionnaire for inclusivity in global research. (DOCX)

S2 File The survey questionnaire. (DOCX)

S3 Table Potential major herb-drug interaction. (DOCX)

S4 Table Potential herb-herb interaction. (DOCX)
