# Supplementary material for: Herbal medicine use disclosure, database-flagged potential herb-drug interactions, and inter – interaction database concordance among patients with non-communicable diseases in Vietnam: A multicenter cross-sectional study
Source: PLoS One. 2026 Jul 31;21(7):e0355046. doi: 10.1371/journal.pone.0355046 (PMC13426966; doi:10.1371/journal.pone.0355046)
Supplement: S2 File — (DOCX) [file pone.0355046.s002.docx]

**S2 File. The Survey Questionnaire**

**PARTICIPANT CONSENT FORM**

Dear Sir/Madam,

You are invited to take part in the research study entitled:

**"Herbal Medicine Use and Potential Herb–Drug Interactions Among Patients with Non-Communicable Diseases in Vietnam."**

The purpose of this study is to understand how patients with non-communicable diseases use herbal medicines and to identify potential herb–drug interactions.

If you choose to participate, the information you provide will be kept strictly confidential and used solely for research purposes. Your participation is entirely voluntary. You are free to decide whether or not to participate, and you may withdraw from the study at any time without any consequences.

If you agree to participate in this study, please fill out the following information:

I have read and been clearly explained the purpose, method and content of the research.

 I agree to participate in the study.

Full name: …………………………………………

Signature: ------------------------------------------------

**Researcher (or person who explains and forms consent)**

I confirm that I have explained to the volunteer the nature and purpose of the study. I have answered the participant's questions. I have answered the volunteers’ questions on the date stated on the consent form.

Full name: .........................................................

Signature: ---------------------------------------------

**SURVEY QUESTIONNAIRE**

**Participant code: ……………………….. Date of data collection: ………./………/………..**

| **A. SOCIALDEMOGRAPHIC** | | | | | | | | | | | | | | |
| --- | --- | --- | --- | --- | --- | --- | --- | --- | --- | --- | --- | --- | --- | --- |
| A1 | Where do you live | | | | 1= Urban  2= Rural | | | | | | | | | |
| A2 | Gender | | | | 1=Male 2=Female | | | | | | | | | |
| A3 | Year of birth | | | |  | | | | | | | | | |
| A4 | What is your education level? | | | | 1= None 2= Primary school  3= Secondary school  4= High school  5= Bachelor’s degree  6= Postgraduate’s degree | | | | | | | | | |
| A5 | What is your monthy income? (Vnd) | | | | 1= ≥ 5 milions  2= < 5 milions  3= No income | | | | | | | | | |
| A6 | What is your occupation? | | | | 1= Retired  2= Worker  3= Business  4= Housework  5= Officer  6= No job  7= Other (Specify……………….) | | | | | | | | | |
| **B. MEDICAL HISTORY** | | | | | | | | | | | | | | |
| B1 | How do you self-assess your current health status? | | | | 1= Poor 2= Neither 3= Good | | | | | | | | | |
| B2 | History of allergy | | | | 1=Yes (Specify: …………………………………… 2=No | | | | | | | | | |
| B3 | What are your chronic diseases? | | | | ……………………………………………………………..  ……………………………………………………………..  ……………………………………………………………..  ……………………………………………………………..  ……………………………………………………………..  …………………………………………………………….. | | | | | | | | | |
| B4. List of your conventional medications | | | | | | | | | | | | | | |
| Name of drug | | | For how long | | | Reason to use | | | | | Fequency of dose | | | |
|  | | |  | | |  | | | | |  | | | |
|  | | |  | | |  | | | | |  | | | |
|  | | |  | | |  | | | | |  | | | |
|  | | |  | | |  | | | | |  | | | |
|  | | |  | | |  | | | | |  | | | |
|  | | |  | | |  | | | | |  | | | |
|  | | |  | | |  | | | | |  | | | |
|  | | |  | | |  | | | | |  | | | |
|  | | |  | | |  | | | | |  | | | |
| **C. LIFESTYLE** | | | | | | | | | | | | | | |
| C1 | Do you use tobacco? | | | | 1= Never  2= Former smoker  3= Current smoker (…… cigarettes/day) | | | | | | | | | |
| C2 | Do you do physical activity? | | | | 1= No 2= Do sometimes  3= Do regularly (…………………. mins/day) | | | | | | | | | |
| C3 | Do you drink alcohol? | | | | 1= Never  2= Former consumer  3= Current consumer (amount:…… cups,cans,glasses/day) | | | | | | | | | |
| **D. PATTERN OF HERBAL MEDICINE USE AND POTENTIAL HERB-DRUG INTERACTION** | | | | | | | | | | | | | | |
| D1. Herbs/herbal medicines you have used in **the last 12 months?** (Name-Form) | | D2.  Do you **currently use** this product? | | D3. Please indicate the main reason that applies to your last use (Check only one) | | | | | D4.  How do you feel after using? | | | | | D5. How do you use? |
|  |  | Yes | No | For an acute illness condition | | To treat your chronic condition | To improve well-being | Other (specify) | Better | Worse | | No change | I don’t know |  |
|  | |  |  |  | |  |  |  |  |  | |  |  |  |
|  | |  |  |  | |  |  |  |  |  | |  |  |  |
|  | |  |  |  | |  |  |  |  |  | |  |  |  |
|  | |  |  |  | |  |  |  |  |  | |  |  |  |
|  | |  |  |  | |  |  |  |  |  | |  |  |  |
|  | |  |  |  | |  |  |  |  |  | |  |  |  |
| D6 | Where do you take the HM most often? | | | | 1= Community pharmacy  2= Folk remedy shop 3= In your own garden 4= Provided by your friends/families  5= Other ……………………………………………………… | | | | | | | | | |
| D7 | Which source do you get HM information? | | | | 1= Family members/Friends 2= Health personel in healthcare facilities 3= Mass media (TV, newspaper, radio, magazine)  4= Social media (Facebook, Tik Tok) 5= Traditional practitioners 6= Self-administration  7= Other ……………………………………………………… | | | | | | | | | |
| D8 | What type of HM information you look for? | | | | 1= Efficacy and benefit  2= Side effect and interaction  3= How to use properly  4= Other ……………………………………………………… | | | | | | | | | |
| D9 | Do you ever consult your doctors or pharmacists about HM use? | | | | 1= Never  2= Sometimes  3= Frequently  4= Always | | | | | | | | | |
| D10 | Reason for not consulting your doctors or pharmacists about HM use? | | | | 1= Not being asked by HCPs 2= HCPs may deny the HM use 3= HM is natural so safe to use 4= HM has no influences on conventional drug using (self-jugdement) 5= Other……………………………………………………… | | | | | | | | | |
| D11 | Do you ever consult your doctors or pharmacists about the potential drug-HM interaction? | | | | 1= Never  2= Sometimes  3= Frequently  4= Always | | | | | | | | | |
| D12 | Do you ever search for information about potential drug-HM interaction? | | | | 1= Never  2= Sometimes  3= Frequently  4= Always | | | | | | | | | |
| D13 | Do you ever experience any side effect when using HM? | | | | 1=Yes (Specify: which HM…..................................Side effect...............................)  2=No  3= I don’t know | | | | | | | | | |

- The End -
