# Supplementary material for: Herbal medicine use disclosure, database-flagged potential herb-drug interactions, and inter – interaction database concordance among patients with non-communicable diseases in Vietnam: A multicenter cross-sectional study
Source: PLoS One. 2026 Jul 31;21(7):e0355046. doi: 10.1371/journal.pone.0355046 (PMC13426966; doi:10.1371/journal.pone.0355046)
Supplement: S3 Table — (DOCX) [file pone.0355046.s003.docx]

**S3 Table. Potential herb-drug interaction (Major severity)**

| Common/Local name used by patients | Interaction drugs | Quality of documentation | Database | Description of interaction | Management |
| --- | --- | --- | --- | --- | --- |
| Gingko Biloba | Ketoprofen | Good | Lexicomp | Ginkgolide B may inhibit platelet activating factors (PAF) induced platelet aggregation | Avoid concomitant use of ginkgo and NSAIDs. If both agents are taken together, frequently monitor the patient for bleeding time and signs and symptoms of excessive bleeding to determine if platelet function has been adversely affected by ginkgo. |
| Sage | Prednisolone | Good | Micromedex | Corticosteroids (Systemic) may enhance the adverse/toxic effect of Acetylcholinesterase Inhibitors. Increased muscular weakness may occur | Monitor for exacerbation of muscular weakness. Dose reduction or tapering of anticholinesterase agents may be beneficial in the setting of high-dose steroid use. Initiation of high-dose steroids in moderate-to-severe myasthenia patients should be undertaken only in the hospital setting to allow close monitoring and respiratory support, if necessary. |
| Turmeric | Aspirin | Good | Lexicomp | Additive effects on hemostasis; duplication of therapy | Curcumin has antiplatelet activity Coadministration of antiplatelet agents increases the risk of bleeding due to an additive effect. If concomitant use occurs, promptly evaluate any signs or symptoms of blood loss. |
| Turmeric | Clopidogrel | Good | Lexicomp | Additive effects on hemostasis; duplication of therapy | Curcumin has antiplatelet activity Coadministration of antiplatelet agents increases the risk of bleeding due to an additive effect. If concomitant use occurs, promptly evaluate any signs or symptoms of blood loss. |
