# Supplementary material for: Herbal medicine use disclosure, database-flagged potential herb-drug interactions, and inter – interaction database concordance among patients with non-communicable diseases in Vietnam: A multicenter cross-sectional study
Source: PLoS One. 2026 Jul 31;21(7):e0355046. doi: 10.1371/journal.pone.0355046 (PMC13426966; doi:10.1371/journal.pone.0355046)
Supplement: S4 Table — (DOCX) [file pone.0355046.s004.docx]

**S4 Table. Potential herb interaction**

| Common/Local name used by patients) | Interaction Herbs | Interaction severity | Database | Description of interaction | Management |
| --- | --- | --- | --- | --- | --- |
| Cinnamon | Dong quai | Moderate | Medscape | cinnamon and dong quai both increase anticoagulation | Use Caution/Monitor. |
| Cinnamon | Ginkgo Biloba | Moderate | Medscape | cinnamon and ginkgo biloba both increase anticoagulation | Use Caution/Monitor. |
| Cinnamon | Mistletoe | Moderate | Medscape | cinnamon increases and mistletoe decrease anticoagulation. | Effect of interaction is not clear, use caution |
| Cinnamon | Panax ginseng | Moderate | Medscape | cinnamon and panax ginseng both increase anticoagulation. | Use Caution/Monitor. |
| Cinnamon | Tumeric | Moderate | Medscape | cinnamon and turmeric both increase anticoagulation. | Use Caution/Monitor. |
| Dong quai | Mistletoe | Moderate | Medscape | dong quai increases and mistletoe decreases anticoagulation. | Effect of interaction is not clear, use caution. Use Caution/Monitor. |
| Dong quai | Ginkgo Biloba | Moderate | Medscape | dong quai and ginkgo biloba both increase anticoagulation | Use Caution/Monitor. |
| Dong quai | Tanacetum parthenium (Feverfew) | Moderate | Medscape | dong quai and feverfew both increase anticoagulation | Use Caution/Monitor. |
| Dong quai | Panax ginseng | Moderate | Medscape | dong quai and panax ginseng both increase anticoagulation | Use Caution/Monitor. |
| Ginger | Feverfew | Minor | Medscape | feverfew and ginger both increase anticoagulation | N/A |
| Ginkgo Biloba | Panax ginseng | Moderate | Medscape | ginkgo biloba and panax ginseng both increase anticoagulation | N/A |
| Ginkgo Biloba | Garlic | Moderate | Lexicomp | Antiplatelet Effects may enhance the adverse/toxic effect of other Herbal Products with Anticoagulant/Antiplatelet Effects. Bleeding may occur | The concomitant use of herbal products possessing anticoagulant/antiplatelet effects with other herbs or drugs possessing similar properties may increase the risk for bleeding. Monitor for signs and symptoms of bleeding if these agents are combined. |
| Green tea | Feverfew | Minor | Medscape | Unknown | Use caution when combining green tea with herbs that exhibit antiplatelet effects or anticoagulant activity. |
| Green tea | Panax ginseng | Moderate | Medscape | Unknown | Use caution when combining green tea with herbs that exhibit antiplatelet effects or anticoagulant activity. |
| Panax ginseng | Mistletoe | Moderate | Medscape | Panax ginseng increases, and mistletoe decreases anticoagulation. Effect of interaction is not clear, use caution. Use Caution/Monitor. | Effect of interaction is not clear, use caution. Use Caution/Monitor. |
